# On the Nature and Treatment of the Diseases of the Heart; with Some New Views on the Physiology of the Circulation

**Published:** 1838-01

**Authors:** 


### ART. IX.

*On the Nature and Treatment of the Diseases of the Heart; with some new Views on the Physiology of the Circulation.* By JAMES WARDROP, M.D., Surgeon to his late Majesty George IV.—*London*, 1837. 8vo. pp. 120; four Plates.

THE title-page intimating that an author of Mr. Wardrop's powers and great professional acquirements had some new views of the circulation to offer, we took up this book with no little interest and expectation. The first part only has yet appeared, and consists entirely of the physiological observations and speculations which the author has deemed it requisite to prefix to his dissertation on Diseases of the Heart. We are sorry to say that, after an attentive and impartial perusal of these observations, we have formed a considerably lower estimate of their value than the author seems to have done.

In the very commencement of the work we meet with several statements, to the truth of which we cannot assent: such as the total want of repose in the muscular fibres of the heart; the existence, in the composition of this organ, of "a considerable quantity of the yellow fibro-cartilaginous substance, similar to the fibrous coat of the arteries," &c. But we pass these by, in order to notice doctrines which Mr. Wardrop considers as new, and to which he evidently attaches great importance.

The first of these is a particular influence exerted by the muscles over the circulation, to which he gives the name of the *Musculo-cardiac function.* After noticing the effect of muscular contraction on the flow of blood in the veins, he observes,

"But, whilst the pressure caused by muscles during their contraction propels the blood onwards in the contiguous veins, it seems never to have been contemplated what must be the effect of that compression on the adjacent arteries; although these vessels are doubtless alike exposed to its influence. It will, however, be shown that the effect of muscular contractions, both on arteries adjacent to, as well as on those imbedded in the substance of muscles, must be to compress these vessels, by which compression the flow of blood through them will be necessarily impeded: hence, the contraction of muscles will increase the accumulation of blood within the heart in two ways,—by accelerating the flow of the venous blood to the right heart, and by impeding the transit of the arterial blood from the left heart." (P. 18.)

An instructive case related by Mr. Hyslop, of a lady in whom syncope was arrested by compressing the brachial arteries, is brought forward to illustrate the stimulant effect on the heart, produced indirectly by accumulation of the blood in its left cavities. Various proofs are adduced in support of the doctrine of the muscular compression of the arteries.

"If the radial artery of a person who is powerfully contracting the muscles of the arm be examined, its pulsations are soon found to become feeble, and at last they are scarcely perceptible; whilst, the moment the muscles are relaxed, the artery is perceived to beat with its natural force. By the same experiment it may also be shown that the effect of impeding the transit of the blood in the arteries is to increase the action of the heart; it being found that, in proportion as the strength of the pulsations of the radial artery diminishes, so is the impulse of the heart increased." (P. 20.)

This experiment requires a little explanation. It might be supposed, on first reading the passage, that the radial artery itself suffers compression; but this cannot be the case; for we have often examined the pulse during the strongest contractions of the muscles of the forearm, and found no change whatever, either in its force or in its volume. Nor do the ordinary actions of the flexors and extensors of the arm, even when very powerful, produce the effect in question. It may be noticed, however, when the biceps and triceps are made to antagonize forcibly, and thus to compress the artery between them; an action which occurs but seldom, and certainly not often enough, in our opinion, to operate importantly on the general circulation. The author proceeds to show that, in those parts which do not require an equable supply of blood, the artery is liable to muscular compression; whilst, in those which do not admit of changes in the quantity, it is protected. Thus, in the limbs, the artery accompanies the vein, in order that both may be exposed to compression, and thus may subserve the musculo-cardiac function. Certainly, as Mr. W. remarks, the importance of the fact of this relative position, though long observed, has not been pointed out; but we must be permitted to doubt the value of his discovery, till he has proved in some more satisfactory manner than by the experiment above noticed, that compression is a frequent occurrence. Mr. W. will pardon us if we venture to conjecture that the principal artery and vein are companions because it is expedient that each should be situated in parts (as in muscular beds,) where they will be defended from external pressure, and be out of the reach of many external injuries. But some organs cannot well bear any interruption in the supply of blood, and therefore they are provided with anastomosing circles, as in the stomach, lips, and iris; all which, but for this provision, would (as Mr. W. thinks,) have been subject to arterial obstruction, from their unceasing movements. We should have thought that the large quantity of blood required by some of these parts would have been an adequate explanation of the anastomoses. The heart, according to Mr. W., is saved from obstruction of its circulation by the position of the coronary arteries; and we quite acquiesce in this explanation of the final cause of the situation of these vessels; for if the trunks had perforated the ventricular substance, they could not have escaped compression. But we really think that the principal arterial trunks, imbedded among the muscles of the limbs, occupy an analogous situation, being guarded in aponeurotic canals, and never passing through the body of a muscle, and therefore similarly shielded from compression.

Like many men who have made discoveries, or imagined that they have done so, Mr. Wardrop often carries his theory to situations seemingly very remote from their legitimate operation, and occasionally applies them in a very fanciful manner. The two following, we conceive, are examples of this sort.

"But an equal and uninterrupted return of the venous blood from the head is of no less importance in the due performance of the brain's functions than a regular supply of arterial blood: hence the importance of that peculiar structure, the unyielding parietes of the veins or sinuses, for warding off the effects of that very compression which is provided in some other veins for the purpose of accelerating the return of the venous blood." (P. 27.)

Mr. W. does not tell us what muscular compression endangers these veins or sinuses unless thus "warded off," and gives us no reasons why the numerous veins of the pia mater and the venæ Galeni do not possess unyielding parietes like the sinuses.

"The vermicular motions of the stomach and intestines during the process of digestion must doubtless have a very considerable influence on the circulation of the blood, both in the veins and in the arteries of these organs; and hence, during the movements of the alimentary canal, we observe an increase in the impulse of the heart, indicated by an increase in the frequency of the pulse. In this respect, the circulation of the blood in the intestinal canal in part resembles that which exists in worms, the almost unceasing movements of these animals being sufficient, as I have before noticed, to circulate their blood, unaided by a heart." (P. 29.)

The author believes that this function "enables us to explain many important phenomena which are constantly occurring in the living body, and which could never have been explained until a knowledge of this function of the muscles had been discovered." The first of these is the remarkable fact that, in passing out of sleep, a man first stretches, then yawns, and then awakes! The movements create an accumulation of blood in the heart, whereby its action is increased; and thus more blood is sent to the heart, which causes the resuscitation of the mental powers. Has Mr. W. never met with persons who awake without any muscular movement except the opening of the eyelids? and with others who, during profound sleep, execute muscular movements amply sufficient, upon Mr. W.'s hypothesis, to arouse the heart? Is it wonderful that, if sleep is a suspension of sensation and motion, the cessation of that state should be often first evidenced by returning movements?

The application of the musculo-cardiac function to the explanation of many familiar facts is often very ingenious, as in the following instances: we wish we could say that it is always as just as it is ingenious.

"If a man be about to make any great exertion, such as running or leaping, he prepares himself, as it were, by first vigorously contracting the muscles of the arms and clenching his hands. For the same reason, when a person is subjected to pain, as that of a surgical operation, he prepares himself to endure it by throwing into action almost all the voluntary muscles, grasping firmly with his hands, and pressing the feet against some resisting body. And when the female, during parturition, is about to make a powerful expulsive effort to assist the uterus in giving birth to the infant, she in like manner throws into violent and long-continued contractions the muscles of the extremities, clenches the jaws, and squeezes with a convulsive effort whatever may be within her reach." (P. 33.)

The first question probably that will occur to the reader of this extract, is,—What good object can be answered by thus forcing the heart to contract by virtue of its distention, allowing that the latter effect is produced? Is a throbbing heart serviceable to a runner or leaper? Does the person who is expecting a surgical operation or the parturient female derive relief from the excited action of the heart? If Mr. W. is right in conjecturing that muscular contraction hurries the blood *away from the muscles* through the veins, and prevents *its access to them* by the arteries, in order to stimulate the heart, we again ask, what object does this stimulation achieve? It appears, contrary to all analogy, that the blood should be sent impetuously to all parts but those which from their vigorous action would seem to need it most. If Mr. W. had found out that persons about to put their brains to unusual exercise, begin by clenching their hands, &c. one might discern a good reason for it in the hypothesis that they thus furnish the thinking organ with a plentiful supply of blood. But setting aside this difficulty arising out of the final cause (which we should have less insisted upon, but that the author devotes himself particularly to this description of causes,) a more natural explanation, if we are not mistaken, may be found for the actions in question. The runner, the leaper, and the cricket-player, exercise their muscles, before their efforts, not to quicken the heart's action, but to bring the motory organs into free play and readiness, as well as to prepare the respiratory apparatus for the change about to take place in the circulation during the exertion. The person suffering or about to suffer pain grasps with his hands and presses with his feet, for the same purpose as he bites his lips; namely, to produce a new sensation *counter-irritative* to the pain. The strivings of the parturient woman have a more complex operation.

We have not space for adverting to the other illustrations of the new function. We may only observe, in passing, that the argument apparently most favorable to the idea of arterial obstruction forms the conclusion of the present section. It is the provision with which certain animals are endowed against the compression of arteries. Thus, in the feline tribe the brachial artery passes through a bony canal in the humerus. But this arrangement appears to us intended to protect the vessel from "pressure from without;" that, for instance, encountered in climbing and in clasping the prey with the anterior extremities. The same arrangement is found in the tardigrade animals which remain suspended for a long time to the branches of trees. In the shark, the aorta runs along a bony channel in the caudal vertebræ, to avoid compression from the powerfully muscular tail. This distribution is not peculiar to the shark or to strong-tailed fishes; but supposing it to be so, would the vessel be liable to this accident from the muscles themselves or from the altered form of the whole organ?

We shall only remark, in addition to what has been stated, that the difference in the structure of the arteries and the veins seems to hint that they are not likely to be assisted in their circulation by the same means, and that while the yielding tissue of the latter, and their valvular provision in parts of the body where they are liable to muscular compression, explain the influence of this action on their function, the firm resistant coats of the former and the absence of any apparatus capable of giving the right direction to the flow of blood in their channels, certainly do not indicate any fitness for the reception of the supposed influence. Another anatomical difficulty occurs to us:—is the structure of the aortal valves at all fitted to cope with such a strain as must be exerted upon them during the musculo-cardiac function? We suspect that they have quite enough to do in withstanding the reflux occasioned by the arterial elasticity; and, at all events, we might naturally expect them to be far stronger than those of the pulmonary artery upon which no muscular effort can directly operate.

To the framer or the supporter of the musculo-cardiac hypothesis, the following question must very soon arise: If the blood, while it is thus pouring into the right heart from the veins, is not allowed to issue in corresponding quantity from the left, and is therefore prevented from entering into the latter, what becomes of it when thus rudely expelled from the one and refused admittance into the other? The answer is presented in another discovery or invention of our author, that of the *pulmo-cardiac function.*

"If there be only a slight increase in the quantity of blood within the heart, such additional stimulus by increasing the vigour of the heart's movements may, along with the elastic quality of the fibro-cartilaginous portion of its structure, which is placed at the roots of the large vessels, be alone sufficient to equalize the circulation. But if the increased supply of blood to the heart be so considerable that the surplus quantity cannot be received within its cavities, the lungs are then required to lend their assistance. To fulfil this *pulmo-cardiac function,* the structure of the lungs is admirably adapted. The pulmonary vessels, being imbedded in a soft and yielding substance, are susceptible of various degrees of distention, so that they readily give way for the reception of any surplus quantity, whether of venous or of arterial blood, and retain it until it can be received within the heart. The structure with which the lungs are endowed to enable the air-cells to accommodate themselves to those differences in the quantity of air which take place during respiration, also enables the pulmonary vessels in their turn to accommodate themselves for the reception of the various quantities of blood which may be impelled into them. A function is therefore performed by the respiratory organs which is quite unconnected either with arterialization of the blood; with aiding the return of the venous blood to the right heart, or with assisting the circulation in the pulmonary vessels; and when it is considered how often and by what slight causes the current of the arterial blood is impeded in leaving the left heart, and how frequently the return of the venous blood is accelerated to the right heart, this pulmo-cardiac function must be regarded as one of primary importance. It exercises, as might be expected, a remarkable influence on the function of respiration, for whenever the egress of the blood from the left heart is impeded by muscular contractions, and the exit of the systemic blood from the lungs into the left auricle is interrupted, the velocity of the blood in the veins will also be increased, and venous blood congested in the pulmonary arteries. Thus the blood being accumulated in the pulmonary arteries and veins, as well as in both hearts, respiration becomes quickened, and its frequency increased in proportion to the degree of impediment which is offered to the distention of the air cells; and the respiration will continue quickened until the circulation be equalized, the heart become tranquil, and the congested lungs relieved of all surplus quantity of blood." (P. 47.)

To us it appears unfortunate that, if the lungs are to play the part of a diverticulum sanguinis, they do not remain passive and allow the blood to accumulate, till the right heart is less disposed to impel, and the left in a better condition to admit. Instead of this, the respiration is quickened, a greater quantity of venous blood is converted into arterial, and pours on towards the left heart, the excitement of which distributes it (in spite of the musculo-cardiac function,) in proportionate quantity to the arteries, as may be lamentably proved in the subject of an aneurismal tumour. We suspect that pulmonary congestion is not so *normal* a condition as to be fitly denominated pulmo-cardiac function. Mr. W. is aware that diving animals are furnished with dilatations of the vena cava, but we might not unreasonably have expected, upon his hypothesis, to have found, in these instances, some convenient sacs at the extremities of the pulmonary arteries also.

The author offers a curious rationale of the method of "training" or "putting a person in wind." He begins with the position that full inspirations by distending the air-cells retard the pulmonary circulation; a position founded upon an observation by Sir E. Home, that "when the arteries of a sheep's lung were injected, the injection returned very readily by the veins; but when the air-cells were previously distended, this did not take place, and the injection could not be forced into the pulmonary veins." But it is one thing to have the lungs distended with air, which produces a change in the blood essential to the capillary circulation, and acting probably by the determination of molecular movements in the fluid itself, irrespectively of the containing parts; and it is another thing to fill the cells with mercury and inject wax into the vessels. The notion, at all events, is the very opposite to that which Haller adopted in explaining the physiology of asphyxia; and to Sir E. Home's experiments we may oppose those of Magendie, which demonstrate that the pulmonary circulation is actually promoted by inspiration. Presuming, however, on the truth of the above statement, Mr. W. infers that "training" consists in acquiring such a control over the breathing as to limit the inspirations, and thus to prevent such a distention of the air-cells as would impede the passage of the blood into the pulmonary veins. The author seems here scarcely consistent with himself; for, if the left heart is struggling with the muscles during their exercise, its difficulties must be rather increased by a greater influx of blood from the pulmonary veins. The greater the stasis in the capillaries of the lungs, the more perfect would be the pulmo-cardiac function!

But, that control over the respiration is much needed during great exertions, and that the inspirations are limited, cannot well be denied.

"Hence in running a race it is found essential that the mouth be kept shut, a sufficient quantity of air entering by the nostrils for the arterialization of the blood, and experience having taught that if any additional quantity is inspired by the mouth, the proper balance between respiration and circulation is more or less destroyed, and the person obliged to discontinue the effort. The common practice, when running, of putting a pebble in the mouth, by the effort of retaining it, keeps the mouth closed. An animal at full speed, it will also be observed, has the mouth always kept shut, either until his muscular powers begin to be exhausted, or when from alarm he inspires by the mouth; so that, whenever an animal which is pursued opens its mouth, it is well known that he cannot long sustain his speed. In like manner for limiting the inspirations not only does the man who runs a race keep his mouth shut, but he also places his arms close to his sides, with the fore-arms in a state of flexure, firmly contracting all the muscles. Artisans also whose avocations require a great muscular effort, are in the habit of tying a belt round the waist; thus preventing too great an expansion of the chest; and when the sailor prepares himself for battle, in order the more powerfully to exert himself at the guns, he ties a handkerchief firmly round the waist, by which, during the excitement of the fight as well as the powerful muscular exertions which he is compelled to make, the movements of the respiratory apparatus are confined within certain limits, and he is prevented from making such full inspirations as would disturb the balance of the circulation within the chest." (P. 59.)

We do not think that these facts are elucidated by Mr. W.'s views. The influence of training is of a far more complex description than would appear from the author's shewing, and involves the consideration of the irritability of the heart, and of the whole nervo-muscular apparatus of respiration. A sudden or unusual exertion occasions a sense of constriction of the chest, which prompts the accessory muscles to active play, and is soon followed by palpitation. Now the quick movements of the thoracic parietes, under the action of the accessory muscles, are quite incompatible with the exertion of other muscles which require the chest for a fulcrum all but immoveable. Panting therefore soon brings the runner to a stop. The respiratory perturbation must be restrained, and this may in a great measure be accomplished by one who possesses such a command over the accessory muscles as prevents their obeying the stimulus of the sensation. Practice will give this degree of command; but the training comprehends a number of processes, all calculated to lessen irritability, and to place the organs of circulation and respiration in a state the very reverse of what we see in hysterical patients, whose heart and respiratory muscles are thrown into convulsive action by an amount of exertion that would have no such effect upon a person of less nervous mobility. The individual trained, however, has diminished even the ordinary irritability, and he manifests in his feats of strength the same superiority over the unpractised though healthy performer, as we noticed in the latter over the hysterical subject. But the gymnastic, however well-prepared, is always cautious to begin his exertions slowly, so as to accustom the heart to the increase of its natural stimulus, and the lungs to a greater accession of blood. The use of limiting the inspirations by the methods enumerated in the passage last cited, we apprehend to be partly to diminish the movements of the chest, and partly to counteract the effect of the muscular exertion on the venous circulation; for we do not doubt that inspiration favours the flow of blood towards the right side of the heart. But it must be borne in mind that although the inspirations are diminished in extent, they are increased in frequency.

Mr. W. next employs himself in unfolding the purposes answered by such phenomena as laughing, crying, sobbing, sneezing, &c.; and although we do not completely accord with his views, we must admit them to be ingenious. The following remarks upon sea-sickness are also ingenious.

"It is from a want of the power of adjustment, or cooperation in the respiratory and circulating organs, by which we can explain sea-sickness. Dr. Wollaston observed, that in those who did not suffer sickness at sea, their respiration was altered. In waking from very disturbed sleep, he found that his respirations were not made with the accustomed uniformity, but were interrupted by irregular pauses, with an appearance of watching for some favorable opportunity to make the succeeding effort, and it seemed as if the act of inspiration were in some manner to be guided by the tendency of the vessel to pitch with an uneasy motion." * * *

"'The mode,' says Dr. Wollaston, 'by which I afterwards conceived that this action could primarily affect the system, was by its influence on the motion of the blood; for, at the same instant that the chest is dilated for the reception of air, its vessels become also more open to the reception of the blood, so that the return of blood from the head is more free than at any other period of a complete respiration. On the contrary, by the act of expelling air from the lungs, the ingress of blood is so far obstructed, that when the surface of the brain is exposed by the trepan, a successive turgescence and subsidence of the brain is seen in alternate motions with the different states of the chest.'* Dr. Wollaston's views of the cause of sea-sickness accord with the means that are usually resorted to for its relief. One mode is placing the body in such a position, that the weight of the column of blood in the head shall be diminished as much as possible during the 'pitching' of the vessel. The other is by modifying the respiration, to prevent congestion within the head. The horizontal position, and placing the head as much as possible to the ship's centre, mitigates the sickness according to the first principle; whilst by the common practice of tying a belt firmly round the waist, or by keeping the mouth shut, and thus limiting the expansion of the parietes of the chest, respiration is so regulated, that the balance of the circulation within the head and chest is preserved." (P. 72-4.)

Several sections are devoted to illustrations of the reciprocal influence of the heart and the brain, and of the heart and the digestive organs; to the blood, and to the pulse; but we find nothing in them to detain us either for exception or for particular commendation. The "Impulse" and "the Sounds of the Heart," have each little more than a page allotted to them. Upon the causes of the latter, we have only the following unsatisfactory paragraph:

"Much has been written and many unnecessary and cruel experiments have been made on living animals, in order to find out the causes which produce the sounds of the heart. But, as Magendie has justly observed when alluding to the discordant opinions on this subject, 'the results of physiological enquiries have not been in proportion to the number of authors who have written on the subject; on the contrary you are disturbed by contradictions of the most glaring nature, or bewildered between a mass of theories and explanations which have no existence but in the minds of the inventors.'" (P. 100.)

Certainly "much *has* been written," and Mr. Wardrop might have relieved the extreme barrenness of his remarks upon this subject by quoting from the works of Williams, Hope, and Bouillaud, and from the highly valuable reports of the committees appointed by the British Association of Science to investigate the causes of the sounds of the heart. We infer that Mr. W. has not paid sufficient attention to the researches of these physiologists, or he would not have been misled by the depreciating observations of M. Magendie, who, however deserving of consideration his dicta may be in other departments of physiology, has so signally failed in this, as to be the last authority that we should have thought to find quoted on the present occasion. We shall be curious to see whether Mr. W. will be able to place his views of the diagnosis of diseases of the heart on a level with the knowledge of the day, if he abstains from referring to the physical states of the organ on which its normal sounds depend.

We conclude our notice of this treatise by remarking that, although it exhibits considerable ingenuity of speculation, we cannot point to any new fact of any consequence that it has established respecting the action of the heart, nor to any such views of facts previously discovered as would seem to have made it worth the author's while to publish them separately. It may be hoped, however, that the forthcoming part, on diseases of the

* Philos. Trans. 1815.

organ will present so rich a store of facts, gathered from the author's experience, as to make us only regret that it was not at once affixed to the physiological introduction; because we might then have passed over in silence what was deficient in the latter, for the sake of the more valuable contents of the former. It is but just to add, that the present little work is written with much clearness and simplicity, and is really very interesting. We have felt much regret in being obliged to differ so frequently with a man of no ordinary talents, as the author proves himself to be, even when most in the wrong.

---

### Art. X.

*Mémoires de la Société Médicale d'Observation, de Paris.* Tome premier. —*Paris,* 1836. 8vo. pp. 495.

*Memoirs of the Medical Society of Observation, of Paris.* Volume Ist. —*Paris,* 1836. 8vo. pp. 495.

